# 3D Textural, Morphological and Statistical Analysis of Voxel of Interests in 3T MRI Scans for the Detection of Parkinson’s Disease Using Artificial Neural Networks

**DOI:** 10.3390/healthcare8010034

**Published:** 2020-02-07

**Authors:** Sabyasachi Chakraborty, Satyabrata Aich, Hee-Cheol Kim

**Affiliations:** 1 Department of Computer Engineering, Inje University, Gimhae 50834, Korea; c.sabyasachi99@gmail.com; 2 Institute of Digital Anti-Aging Healthcare, Inje University, Gimhae 50834, Korea; 3 u-HARC, Inje University, Gimhae 50834, Korea

**Keywords:** Parkinson’s disease, neurodegeneration, MRI, 3D, texture, morphology

## Abstract

Parkinson’s disease is caused due to the progressive loss of dopaminergic neurons in the substantia nigra pars compacta (SNc). Presently, with the exponential growth of the aging population across the world the number of people being affected by the disease is also increasing and it imposes a huge economic burden on the governments. However, to date, no therapy or treatment has been found that can completely eradicate the disease. Therefore, early detection of Parkinson’s disease is very important so that the progressive loss of dopaminergic neurons can be controlled to provide the patients with a better life. In this study, 3T T1-MRI scans were collected from 906 subjects, out of which, 203 are control subjects, 66 are prodromal subjects and 637 are Parkinson’s disease patients. To analyze the MRI scans for the detection of neurodegeneration and Parkinson’s disease, eight subcortical structures were segmented from the acquired MRI scans using atlas based segmentation. Further, on the extracted eight subcortical structures, feature extraction was performed to extract textural, morphological and statistical features, respectively. After the feature extraction process, an exhaustive set of 107 features were generated for each MRI scan. Therefore, a two-level feature extraction process was implemented for finding the best possible feature set for the detection of Parkinson’s disease. The two-level feature extraction procedure leveraged correlation analysis and recursive feature elimination, which at the end provided us with 20 best performing features out of the extracted 107 features. Further, all the features were trained using machine learning algorithms and a comparative analysis was performed between four different machine learning algorithms based on the selected performance metrics. And at the end, it was observed that artificial neural network (multi-layer perceptron) performed the best by providing an overall accuracy of 95.3%, overall recall of 95.41%, overall precision of 97.28% and f1-score of 94%, respectively.

## 1. Introduction

Parkinson’s disease (PD) is the second most common neurological disorder among elderly people with ages above 65 years. This disease affects the brain cells, which in turn creates cognitive disorders as well as movement disorders in healthy human beings. As the disease is progressive in nature, early detection and monitoring can save a huge amount of healthcare costs. As the population of old age people is increasing and also in the future, it will increase exponentially, therefore it is necessary to find suitable methods for the detection of neurodegenerative diseases at a very early stage [1,2,3]. One of the widely used diagnostic tools, known as Magnetic Resonance Imaging (MRI), has been known for providing anatomical details of brain tissues that could be used for early detection of PD. Although MRI provides a lot of important details, it is really difficult for the human eye to detect the intrinsic details and the heterogeneous properties of certain tissues of the region of interests (ROI’s) [4]. With the advancement of technology, computer-aided diagnostic tools have been considered very effective for the detection and diagnosis of diseases as it provides morphological details about different tissues of the ROIs.

The researchers in the past found that texture analysis of tissue and cellular images can be used for the detection and diagnosis of multiple diseases. Its application is huge starting from segmentation of the identified anatomical structures at the ROIs to the classification of tissues. The quantification of grey-level patterns and pixel inter-relationship within an image, which can measure the heterogeneities in the tissues can also be performed using texture analysis using a variety of techniques. It has been found that the textual patterns are different for different image areas, which are sometimes unnoticeable to the human eyes [5]. In the past researchers have used texture analysis for segmentation and classifications of lesions and tissues which are widely used for diagnosis of critical diseases. In recent years the texture analysis has been widely used in neurological applications [6]. Some of the applications of texture analysis in neurological disorders include differentiation between different types of tumors, classification of diseases like Alzheimer’s, Friedreich’s ataxia, etc. [7,8,9,10].

Traditionally 2D textural analysis is most commonly used for any application, but in the recent years 3D textural analysis also received a lot of attention because 3D analysis provides more discriminative information compared to 2D analysis. In 2D texture analysis, the individual pixel of interest (POI) has eight neighbor pixels that can be analyzed in four directions that are independent of each other. Whereas in 3D texture analysis, the individual voxel of interest has 26 neighbor voxels that can be analyzed in the 13 directions that are independent of each other [11]. In recent years machine learning and deep learning techniques have been widely used for medical applications especially to improve the clinical practice [12]. Machine learning and deep learning techniques are widely used for improving the efficiency in the radiological practices and assisting the doctors to take decisions in a faster way [13]. Therefore, the proposed study puts forward a 3D Texture, morphological and statistical analysis of the Voxel of Interests for the detection of Parkinson’s disease using machine learning algorithms.

The structure of the paper is organized as follows: Section 2 describes the related work of the study and the past works that have an alignment with the present study. Section 3 puts forward the specification of the data that was collected from the Parkinson’s Progression Markers Initiative (PPMI) database and the atlases that were used for the preprocessing of the acquired MRI scans followed by atlas-based segmentation of eight subcortical structures of the basal ganglia which are prone to neuron degeneration. Section 4 describes the methodology of the complete work and also extensively discusses the feature extraction, feature engineering, and feature selection process. Moreover, Section 4 also puts forward the hypothesis for the development of machine learning algorithms and also gives a complete overview of the learning process and comparative analysis. Section 5, plots the results of the complete study and Section 6 discusses the overall implication and usefulness of the study. Finally, the paper is concluded in Section 7.

## 2. Related Work

In the past, texture analysis has been applied to medical imaging and has already shown its potential as a biomarker for discriminating diseases based on healthy tissue and damaged tissues in the ROI. Feng et al. proposed a method that used texture analysis and shapes analysis to model the hippocampus region based on the structural MRI to distinguish mild cognitive impairment (MCI) from Alzheimer’s disease. They have found that their model is performing well by comparing with the state of art models [14]. Martinez-Murcia et al. proposed a method that used texture analysis to find out the structural changes occurred in the different regions of the brain when a person is suffered from Alzheimer’s disease (AD).

In this research, they have focused only on cortical and subcortical parts of the brain and they found that this method could able to provide accuracy up to 81.3% to detect the changes in AD [15]. Sikio et al. proposed a method using texture analysis to detect the structural changes in the brain in PD patients. They have monitored the patient for two years and compared the structural changes with the MRI baseline i.e., taken at the beginning of diagnosis to the MRI after 2 years. They have observed that texture analysis can discriminate against the changes that occurred in the brain and it was significantly related to clinical scores with respect to the severity of the diseases [16]. Betrouni et al. proposed a method that used textural analysis for discrimination PD patients from healthy controls. They have considered six regions of the brain and extracted first-order and second-order features as a part of the textural analysis and their correlation related to cognitive function. They found that these textual features could able to distinguish PD and also help in the diagnosis of PD [17]. Li et al. performed an extensive study regarding the 3D texture analysis of substantia nigra of Parkinson’s disease patients on quantitative susceptibility maps (QSM) and R2* maps. The primary objective of the study was to discriminate PD patients from health control (HC). Therefore, the authors obtained the QSM and R2* maps from the 3T MRI scans from 28 PD and 28 HC using 3D multi-echo gradient-echo sequence. Further, first and second order textural features were obtained from the QSM and R2* maps for discriminating the PD and HC using two tailed t-test. And it was found that first and second order QSM textual features accurately distinguished PD from HC [18].

Zhang et al. proposed a method that used 3D textural analysis for distinguishing AD patients and healthy normal controls by extracting features from the entorhinal cortex and hippocampus regions. They found that 3D features could able to find the difference in the texture of AD patients and normal patients and this proposed method could be helpful for the diagnosis of AD [19]. Lee et al. proposed a method that could predict the progression from the stage mild cognitive impairment (MCI) to AD at the early stage based on the features extracted from the hippocampus, precuneus, and posterior cingulate cortex regions. It was found that the extracted textures from the mentioned three regions could able to predict MCI to AD at an early stage [20]. Li et al. proposed a method that can distinguish AD, MCI, and normal control based on the 3D textural features obtained from the hippocampus region. They found that the pathological changes that occurred in the hippocampus region of the patients could be analyzed using 3D textural analysis and this method could help to diagnose the patients at the early stage of AD [21].

Xiao et al. proposed a method that can classify AD from McEwan normal controls using a multi-feature technique and fused it together by taking the 2D and 3D information of the brains. They have used feature selection techniques to extract optimal feature combinations and that produces better classification performances. They found that the proposed method could help to identify different classes without apparent symptoms [22]. Maani et al. proposed a texture analysis method that used the extracted features on a voxel by voxel basis for the classification of AD and control groups. It was found that the artificial effects of AD and the control group have been detected with high accuracy using this proposed method. The accuracy of the result provided enough support to extend it for other diseases such as cerebral pathology in neurological diseases [23]. Ta et al. mentioned that 3D texture analysis has the potential for the detection of cerebral degeneration which is not easily noticeable for the naked eye. They have found that 3D texture analysis has shown potential and enough reliability that it can be used for longitudinal studies [24].

The above-mentioned literature confirms the success of the analysis of MRI scans for the detection of Neurodegenerative diseases such as Alzheimer’s and Parkinson’s. Moreover, texture, morphological and statistical analysis of ROI for Alzheimer’s disease have been pathbreaking. Therefore the findings of the above-described studies motivated us to design the proposed study for the analysis of the textural, morphological and statistical features of the eight subcortical structures prone to neuron degeneration and responsible for Parkinson’s disease.

## 3. Data Collection and Preprocessing

### 3.1. Data Collection

The data for the study was obtained from the Parkinson’s Progression Markers Initiative (PPMI) database (www.ppmi-info.org/data). PPMI is a landmark, international and multicenter study to determine Parkinson’s progressions biomarkers. All the MRI scans that were selected for the study were performed at the baseline visit. All the scans that were considered for the study were acquired from a similar type of scanner (SIEMENS, Munich, Germany) and also all the MRI scans were based on Magnetization Prepared—Rapid Gradient Echo (MP-RAGE) sequence. The scans were acquired in a time range of 20–30 min and the field of view (FOV) of all the images included vertex, cerebellum, and pons. Moreover, all the scans were further chosen using some specific criteria as mentioned in Table 1.

After applying the filters based on the imaging protocol mentioned in Table 1, a total of 906 MRI scans where chosen from the baseline visit of 906 patients. Out of the 906 patients, 306 were female and 600 were male. The age of all the enrolled subjects was 62.64 ± 9.944. Moreover, the scans were acquired from primarily three groups, namely control, prodromal and Parkinson’s disease. The distribution of all the scans distributed into the research groups as 203 control, 66 prodromal and 637 Parkinson’s disease. For considering a subject to a particular research group certain eligibility criterion was considered which are mentioned in Table 2.

**Figure 1:**
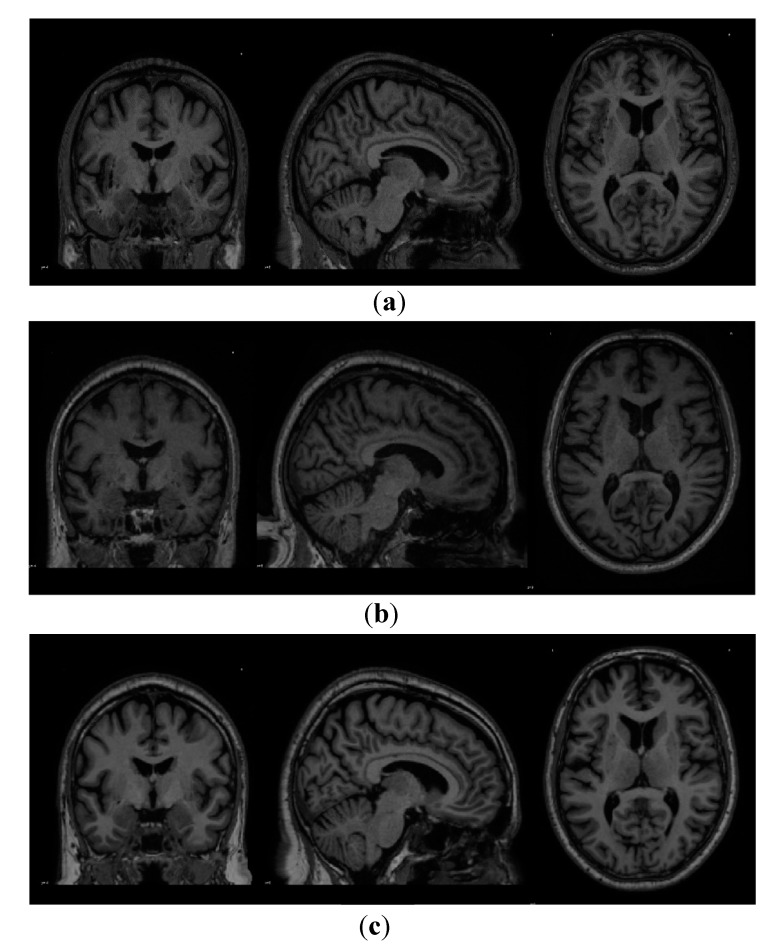
Sample images extracted from the PPMI repository. (**a**) MRI scan of a subject belonging to the control group. (**b**) MRI scan of a subject belonging to the prodromal group. (**c**) MRI scan of a subject belonging to the Parkinson’s disease group.

Figure 1 below plots out the sample MRI Image scans belonging to each research group which was acquired from the PPMI database and Table 3 describes the specifications and the metadata of the acquired MRI scans.

### 3.2. Data Preprocessing

The dataset that was acquired from the PPMI database was collected from multiple study centers across the globe and the scans from different centers had multiple temporal differences. Therefore to maintain a constant spatial tendency between all the scans and also to bring the scans to the same space such as Montreal Neurological Institute (MNI) or Individual Brain Atlases using Statistical Parametric Mapping (IBASPM) [25], an image registration procedure was performed. Image registration is a process that mutates upon a fixed image to find the correct alignment parameters so that an unknown or unseen image can be aligned similarly to the fixed image. In the simplest form, it can be stated that it is a process of aligning two images where one acts as the target image and the other acts as a source image, and the source image is transformed to match the target image. In the present context, the acquired image from the PPMI database is considered as the source image and the target image is an atlas such as MNI or IBASPM.

**Figure 2:**
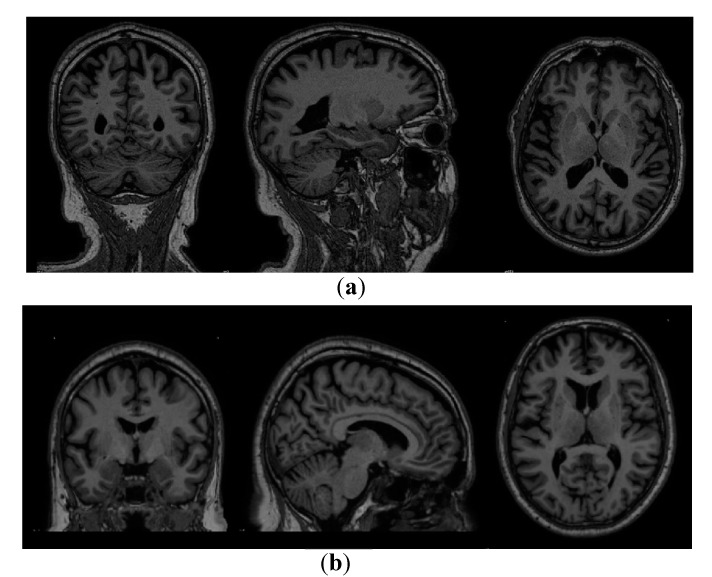
Sample scan pre and post-registration. (**a**) MRI scan before registration (**b**) MRI scan after registration.

The registration of the acquired scans from the PPMI database was performed using the MNIPD25-T1MPRAGE-1 mm atlas developed by Xiao et al. [26,27,28]. Table 4 below describes the specification of the atlas that has been used for the registration. For the registration of the scans, ANTsPy [29] was leveraged which is used for extracting information from complex imaging datasets and is considered to be one of the most effective tools for performing preprocessing on MRI, fMRI, and SPECT scans. The process of registration leveraged a symmetric normalization which performed an affine and deformable transformation on the acquired MRI scans. Figure 2 below shows the scan of a particular image before and after the registration respectively.

## 4. Materials and Methods

**Figure 3:**
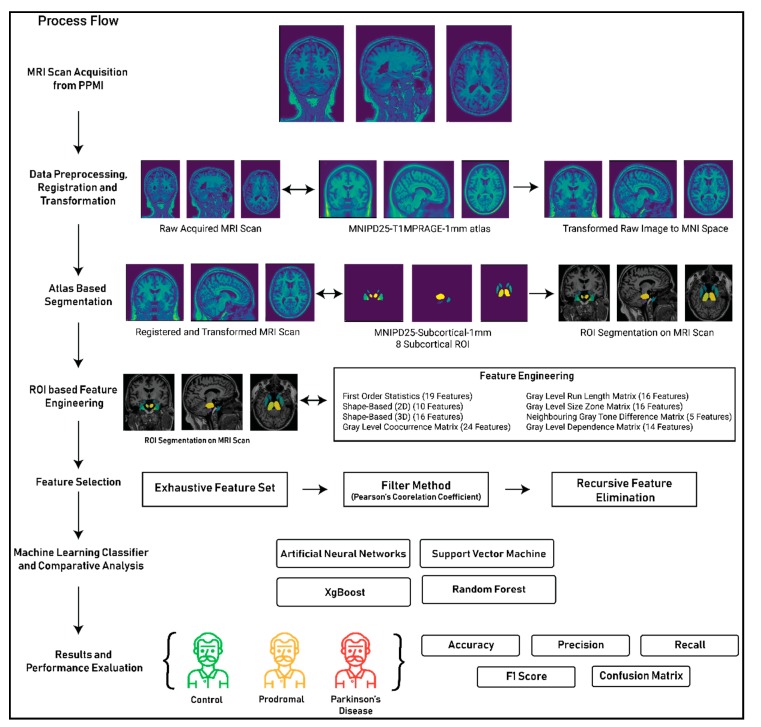
Complete process flow of the study.

The primary proposition of the complete study plots the method of detecting and classifying MRI scans of subjects as control (healthy subjects), Prodromal (stage where the patients do not fulfill the diagnostic criteria for Parkinson’s disease such as bradykinesia or display any motor symptoms but demonstrate signs and symptoms of developing motor symptoms in future) and Parkinson’s disease using textural features. Figure 3 plotted below shows the complete methodology of the performed study and shows the process of leveraging textural features from MRI scans to classify the images as either control, prodromal or Parkinson’s disease.

In the above figure, the complete process for the 3D texture analysis of eight subcortical ROI’s of an MRI scan for the detection of Parkinson’s disease is been demonstrated. The complete process is divided into seven different stages. The first two stages of the process, that is MRI acquisition from the PPMI Database and data preprocessing, registration and transformation, have been described thoroughly in Section 3. The next four stages of the process that are atlas-based segmentation, ROI- based feature engineering, feature selection, machine learning classifier development and performance evaluation of the classifier are described in the following sections. 

### 4.1. Atlas Based ROI Segmentation

**Figure 4:**
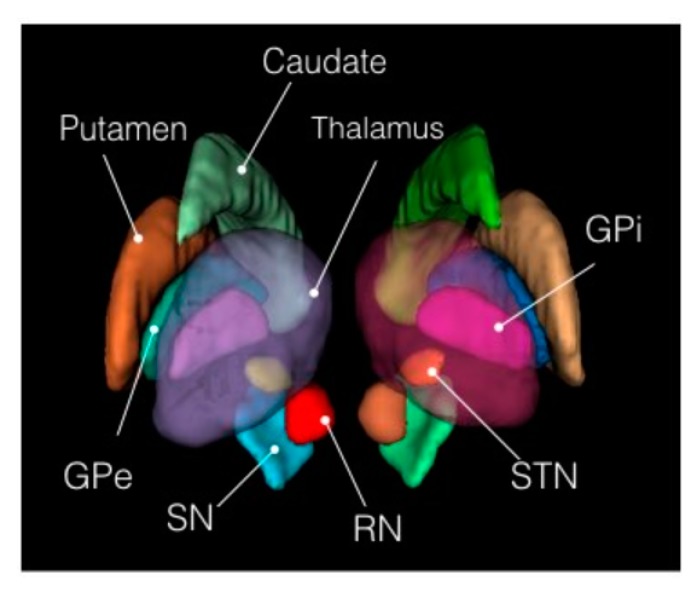
3D view of the eight subcortical structures. Source: http://nist.mni.mcgill.ca/?p=1209.

**Figure 5:**
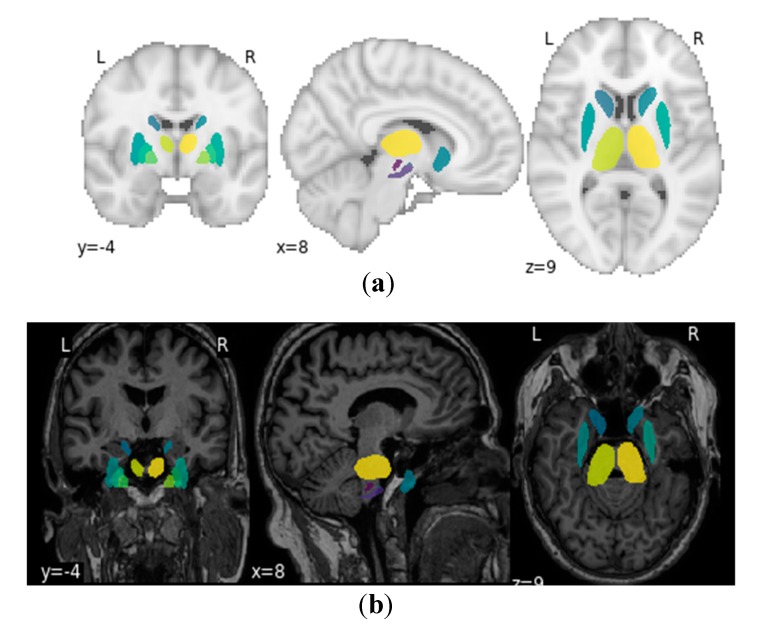
ROI plotting on (**a**) MNI-ICBM-152 template and (**b**) an acquired MRI scan from PPMI.

The study focused on deriving textural features, morphological features and statistical features from the MRI scan for the detection of Parkinson’s disease. The detection of Parkinson’s disease has been studied in previous works [30,31,32,33] where it is shown that anatomical changes occur in the structures of basal ganglia and its adjacent structures due to neurodegeneration which is the primary cause of Parkinson’s disease. The basal ganglia constitute a set of structures that are found at the innermost part of the cerebral hemisphere. The structures that constitute the basal ganglia are the caudate-putamen, global pallidum, substantia nigra and the subthalamic nucleus. Therefore, for fetching the ROIs from the MRI images that are responsible for Parkinson’s disease, atlas-based ROI segmentation was performed on the MRI images acquired in the study. The atlas that was selected for the ROI-based segmentation was MNIPD25-Subcortical-1 mm [26,27,28]. The atlas was developed by manually segmenting eight subcortical structures, namely, caudate nucleus, putamen, globus pallidus internus and externus (GPi & GPe), thalamus, STN, substantia nigra (SN), and red nucleus (RN). Figure 4 below depicts the 3D structure of the eight subcortical structures of the basal ganglia and its adjacent regions. Figure 5 presents the ROIs on the MNI space by plotting it on the MNI-ICBM-152 template [34,35,36] and on a sample MRI scan acquired from the PPMI database.

**Figure 6:**
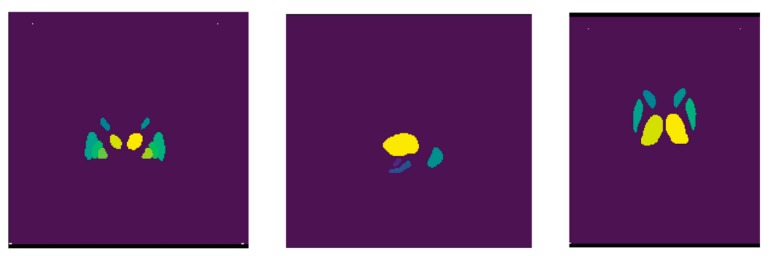
Extracted Voxels from an acquired MRI post segmentation.

The labels of the ROIs of the MNIPD25-Subcortical-1 mm atlas were used for segmenting the acquired MRI scans. The labels with the definition of each label are further described in Table 5. For the segmentation procedure, the voxel indices from the MNIPD25-Subcortical-1 mm atlas were calculated by maintaining the spacing, orientation and slice thickness of the slices. Further, the same indices from the registered and transformed acquired MRI were extracted by similarly maintaining the spacing, orientation and slice thickness of the slice. By following this particular method all the 16 volumetric ROI’s from eight subcortical structures were extracted. Figure 6 below shows a scan which only contains the voxel of interest after performing the segmentation.

### 4.2. ROI Based Feature Engineering

The feature engineering for the study was performed on the segmented 3D voxels from the MRI scans acquired from the PPMI database. The features that were extracted from the segmented 3D voxels were textural, morphological and statistical features [37]. In total 107 features were extracted from the segmented voxels. For the calculation of features, initially, all the 107 features were calculated for each of the 16 subcortical structures/labels mentioned in Table 5. Further, the feature values of a particular feature from all the 16 subcortical structures were aggregated using statistical techniques such a mean, mode and sum to create one feature value for all 16 subcortical structures for each image. In this study, it can be observed that an exhaustive set of features has been computed as compared to the previous studies because we also aim to identify the best features in terms of MRI scans for the prediction of Parkinson’s disease. Table 6 below provides the details of the types of features that were calculated for the feature engineering.

### 4.3. Feature Selection and Machine Learning Classifier Development

In this study, an exhaustive list of features was calculated based on the texture, morphology, and statistics of the 3D voxels of the segmented subcortical structures, but the wide list of features contained a lot of correlation. Moreover, as mentioned previously that choosing the appropriate feature set is also a proposition of the study. Therefore for choosing the right set of features for the machine learning model, a two-level feature selection method was used by leveraging a filter method (Pearson’s correlation coefficient) and a recursive feature elimination method.

After performing correlation analysis on the features, 42 features were rejected based on a Pearson’s correlation coefficient of more than 90%. Further, the remaining 65 features were extracted from the exhaustive feature set for performing the second level feature selection using the recursive feature elimination [39] method. Recursive feature elimination is a greedy optimization algorithm which aims to find the best performing feature subset. It repeatedly creates baseline models and keeps aside the best or the worst performing feature at each iteration until all the features are exhausted. It then ranks the features based on the order of their elimination. Therefore, for our study, a logistic regression model was used for creating the baseline model and for initiating the recursive feature elimination process. Moreover, the recursive feature elimination was also initiated with an argument of fetching 20 best features based on optimum performance of the baseline model in the detection of prodromal and Parkinson’s disease cases, respectively. Moreover, a recursion was also for selecting 10, 30 and 40 best features, respectively, but it was found that selecting 20 best features using recursive feature elimination gave the best result. Table 7 above provides information about the feature that was derived by running the recursive feature elimination and has been arranged in descending order of feature importance.

As the features have been generated and the optimum number of features have been selected from the exhaustive feature set, therefore now the primary motive of the study is the development of the machine learning models for the classification of the MRI scans into three classes namely, control, prodromal, and Parkinson’s disease. The development of machine learning models are very essential for the development of such an automated system for the classification of textural, morphological and statistical features of brain MRI scans for the detection of Parkinson’s disease. Therefore, for the development of the machine learning model, four different machine learning algorithms were used for classification purposes. Also, the usage of four machine learning algorithms for the classification purpose is only required for performing a comparative analysis between the performances of the classifiers. Moreover, for the development of machine learning algorithms, the first this that needs to be determined is the hypothesis of the problem that needs to be solved, so the primary hypothesis that was devised for developing of the machine learning algorithms are as follows:

(1) The recall of Parkinson’s disease class must be 100% and there should not be any mispredictions of the samples belonging to Parkinson’s disease class to any of the other two classes.
(2) For the prodromal class, there must not be any mispredictions of samples belonging to the Prodromal class to the Control class.
(3) The recall of the control class must be more than 85%.

Therefore based upon the above-mentioned hypothesis, the machine learning models were developed. For checking the performance of the machine learning models five different classification performance metrics were leveraged namely, accuracy, recall, precision, f1 score, and confusion matrix. Also, for checking the generalizability of the developed machine learning model 5 split cross-validation was performed. The details about the performance of the cross-validation are described properly in the Results section. Table 8 below provides the information regarding the machine learning algorithms used and also the hyperparameters that was used for the development of the machine learning algorithms.

Moreover as mentioned in the data collection section, the distribution of the MRI scan samples was 203 control, 66 prodromal and 637 Parkinson’s disease, which is considered to be highly imbalanced in nature. The presence of imbalanced data highly affects the performance of the learning models [40,41,42,43,44]. Therefore, presently there are multiple techniques that have been developed to tackle such problems such as the undersampling of the data, the oversampling of the data, synthetic minority oversampling technique (SMOTE) [45], but in the present study, such methods of increasing or decreasing the cardinality of the dataset were not used rather a class weight-based method was utilized [46]. The class weights were basically calculated for each class based upon the number of samples it possesses in the dataset with respect to the total number of samples in the dataset. This method was utilized to create a weighting mechanism for the calculation of the loss function and the class with minority samples are weighted more during the training process. For determining the class weights a specific technique was used which is given as follows:

$$\mathrm{weight\_of\_particular\_class}=\;\left|log\left(\frac{tuning\_parameter\;*\;number\_of\_total\_samples}{number\_of\_samples\_of\_particular\_class}\right)\right|$$

where the tuning_parameter is a constant value that needs to be iteratively tuned and for the study, it was chosen as 0.5. Table 9 below shows the class weights that were chosen for each class.

## 5. Results

The developed machine learning algorithms uncovered pretty astounding and effective results for the detection of Parkinson’s disease from 3D MRI scans. Table 10 below shows the comparative analysis between the four different classifiers that were used for learning the textural, morphological and statistical features of the ROI’s of MRI scans and plots the results of the five split cross-validation which were used for determining the generalizability of the model. The scores mentioned in Table 10 are based on the validation set of each cross-validation split. From the comparative analysis, it was found that the artificial neural network in the second spit outperformed all the other classifiers by giving an overall accuracy, precision, recall and F1 score of 95.3%, 0.9728, 0.9541, and 0.94 respectively. Moreover, having artificial neural networks in the production environment and real-time implementation is also beneficial as it provides support for incremental learning.

**Figure 7:**
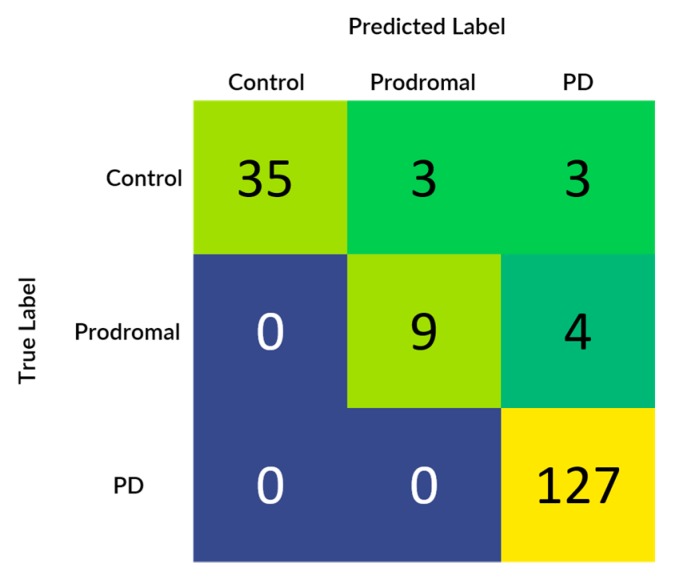
Confusion matrix of artificial neural network at test split 2.

Figure 7 demonstrates the confusion matrix that was generated based upon the results received from the best performing classifier that is artificial neural network (multi-layer perceptron). Also, from the confusion matrix it can be evidently observed that the results completely align with the initial hypothesis which states the recall of the samples belonging to the Parkinson’s disease class needs to be 100%, in prodromal class, there should be no mispredictions in the control class and recall of the healthy class should be more than 85%.

**Figure 8:**
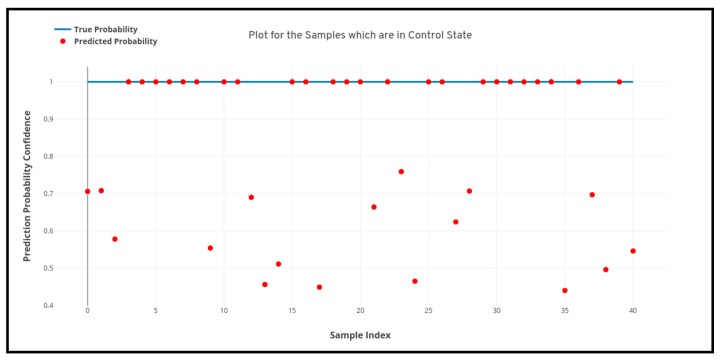
Quantile-quantile plot between the true probability and the prediction probability of the samples belonging to the control class. Probabilistic confidence is 0.82.

**Figure 9:**
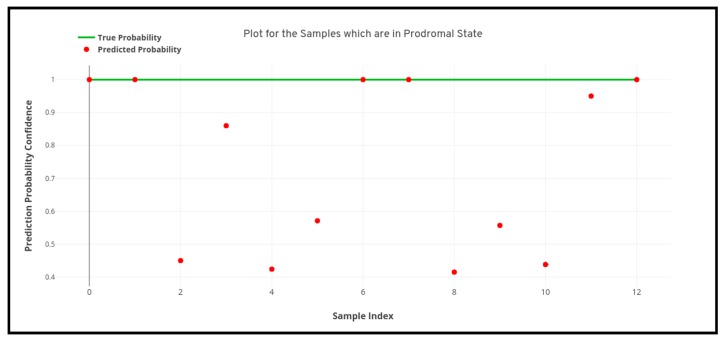
Quantile-quantile plot between the true probability and the prediction probability of the samples belonging to the prodromal class. Probabilistic confidence is 0.641.

**Figure 10:**
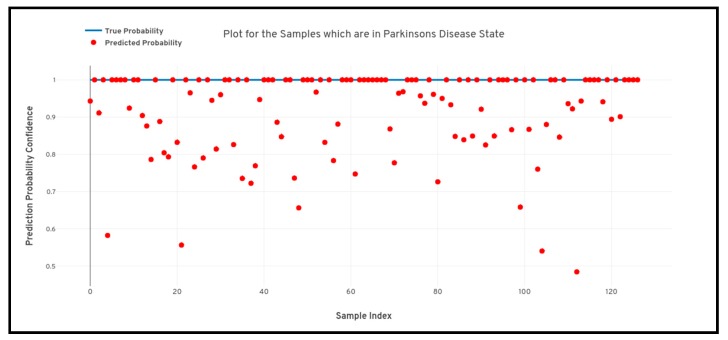
Quantile-quantile plot between the true probability and the prediction probability of the samples belonging to the Parkinson’s disease class. Probabilistic confidence is 0.919.

For the study as we can see above that four different machine learning algorithms were developed for the detection of Parkinson’s disease from 3D MRI scans, but as we can see there remains some inconsistency among the performance of the different machine learning models as the artificial neural network (multi-layer perceptron) model performs the best and the support vector machine performs the worst. Therefore, apart from analyzing just the predictive performance of the machine learning models, uncertainty and prediction confidence analysis also need to be performed for determining the best model. Figure 8, Figure 9 and Figure 10 depicted below shows the quantile-quantile plot (QQ plot) of the prediction probabilities of each class for artificial neural network. In the below-plotted figures, the straight line depicted shows the true probabilistic value (i.e. always 1.0) of a particular sample belonging to a particular class, whereas the red markers show the prediction confidence a particular sample of a particular class.

## 6. Discussion

In this work, a process was developed for classification of the textural, morphological and statistical features derived from 3D voxels of interests of MRI scans for the detection of Parkinson’s disease. In this study, MRI scans were collected from the PPMI database from three different research groups namely, control, prodromal and Parkinson’s disease. The first stage of the work was to identify the ROI’s that are responsible for Parkinson’s disease and are affected during the process of neurodegeneration in older adults. Therefore eight subcortical structures from the MRI scan were chosen for the specific task. The eight subcortical structures are the caudate nucleus, putamen, globus pallidus internus and externus (GPi and GPe), thalamus, STN, substantia nigra (SN), and red nucleus (RN). For the segmentation of the eight subcortical structures from the MRI scans, an atlas-based segmentation was used for extracting the voxels of interest from the MRI scans. The voxels of the acquired MRI scans were registered into the MNI space using affine and symmetric registration to generalize all the acquired MRI scans into a particular space. Further, the MRI scans are subjected to a segmentation routine to fetch only the voxels from the MRI scans which are from the eight subcortical surfaces. Post extraction of the voxels of interest from the ROI regions, the primary motive is to extract the features from voxels of interest.

For the feature engineering process a multitude of features belonging to textural, morphological and statistical domains. The features that were engineered from the 3D arrays of the voxels of interest were first order statistical features, shape-based (2D & 3D) features, gray level cooccurrence matrix features, gray level run length matrix features, gray level size zone matrix features, neighbouring gray tone difference matrix features and gray level dependence matrix features. In total 107 exhaustive features were calculated from the extracted voxels. Now as there were such a huge number of features in the exhaustive list and multiple features were highly correlated with each other. Therefore, the implementation of a feature selection technique for selecting the best features came in as a requirement. For the feature selection process, a two-level selection paradigm was used which consisted of correlation analysis using the filter method (Pearson’s correlation coefficient) followed by recursive feature elimination. In the process of correlation analysis of the features, 42 features were rejected out of 107 exhaustive features and 65 features were selected. The exclusion principle of the features based upon the correlation analysis was performed by considering a correlation coefficient of >90%. After correlation analysis, a routine of recursive feature elimination was performed on the remaining 65 features and the top 20 features were selected based upon the feature importance.

The final segment of the study was concerned about the development of learning algorithms for identifying control, prodromal and Parkinson’s disease from the MRI scans. For the development of the algorithms, a comparative analysis was performed by training four different machine learning algorithms with extracted features. The four different algorithms that were used are artificial neural network, XgBoost, random forest classifier, and support vector machine. Moreover, a 5-split cross-validation was performed across all the classifiers to check the generalizability of the model. From the results, it was observed that the artificial neural network performed the best and gave an overall accuracy of 95.3%, overall recall of 95.41%, the overall precision of 97.28% and f1-score of 94% respectively. Moreover, the artificial neural network model performed robustly by classifying all the samples belonging to Parkinson’s disease correctly with a confidence of 91.9%. Therefore, in the end, the best performing classifier was selected as the final machine learning model for the study.

## 7. Conclusions

In this proposed study, a 3D MRI analysis was performed by leveraging textural, morphological and statistical features from the voxels of interest for the detection of Parkinson’s disease. The study performed an atlas-based segmentation on 3D MRI images to extract eight subcortical structures namely the caudate nucleus, putamen, Globus pallidus internus and externus (GPi and GPe), thalamus, STN, substantia nigra (SN), and red nucleus (RN) and perform feature engineering on the extracted voxels of interest. The engineered features were also subjected to a feature selection method using correlation analysis and recursive feature elimination to fetch the best performing features. Post feature extraction the study also performed a comparative analysis on learning algorithms by leveraging four machine learning algorithms and determine the best performing model using the classification performance metrics. After the development of the learning model it was found that the artificial neural networks performed the best in terms of classifying Parkinson’s disease and also aligned with the initial hypothesis that was developed before initiating the machine learning model development The developed artificial neural network performed the best by plotting an overall accuracy of 95.3%, overall recall of 95.41%, the overall precision of 97.28% and F1-score of 94% respectively.

To conclude, the outcome of the proposed study is very motivating. However, there is still a huge scope of untouched study when it comes to the analysis of the voxels of interest or MRI scans for the detection of Parkinson’s disease. Moreover, this study was purely based on the principles and the scope of 1st generation artificial intelligence where the ground knowledge of the researcher or the developer is used for feature engineering and then the development of the learning model, but with the advent of 2nd generation artificial intelligence which consists of deep learning, reinforcement learning, etc., now the algorithms have the capability to learn the fetch feature representations from the data to map them with a particular outcome. Therefore, in upcoming studies, it is very much recommended that more research should be performed on a particular topic to understand the capability of the artificial intelligence in whole to detect Parkinson’s disease.

## Figures and Tables

**Table 1:** Parameters for choosing MRI Scans from the PPMI Study.

Imaging Protocol	Values
Modality	MRI
Research Group	Control, Prodromal and PD
Visit	Baseline
Acquisition Plane	Sagittal
Acquisition Type	3D
Field Strength	3.0 Tesla
Flip Angle	9 Degree
Scanner Manufacturing	Siemens (MPRAGE)
Pixel Spacing	0.9 mm–1.5 mm (X &Y)
Slice Thickness	1.0 mm
Weighting	T1

**Table 2:** Eligibility Criteria for the Subject to be included in a Research Group.

Research Group	Criteria
Parkinson’s Disease	Patients must have at least two: resting tremor, bradykinesia, rigidity, asymmetric resting tremor, asymmetric bradykinesia. Diagnosis of Parkinson’s Disease for 2 years. Hoehn and Yahr stage I or II Male or Female age 30 Years or Older.
Control Subjects	Male or Female age 30 Years or Older. Not First degree relative to Parkinson’s patient
Prodromal	Male or female age 60 years or older No active clinically significant neurological disorder. Clinical diagnosis of Parkinson’s disease as determined by the investigator.

**Table 3:** Specification of the acquired scans from PPMI.

Image Parameters	Values
Dimensions	256 × 256 × 170–200 pixels
Interslice Gap	0.0 mm
Slice Thickness	1.0 mm
Spacing	1.0 × 1.0 × 1.0 mm
Plane	Sagittal

**Table 4:** Specification of the acquired scans from MNIPD25-T1MPRAGE-1 mm atlas.

Image Parameters	Values
Dimensions	193 × 229 × 193 pixels
Interslice Gap	0.0 mm
Slice Thickness	1.0 mm
Spacing	1.0 × 1.0 × 1.0 mm
Plane	Sagittal

**Table 5:** Labels of each subcortical structure of the MNUPD25-Subcortical-1 mm atlas.

Label	Structure
1	Left red nucleus
3	Left substantia nigra
5	Left subthalamic nucleus
7	Left caudate
9	Left putamen
11	Left globus pallidus external
13	Left globus pallidus internal
15	Left thalamus
2	Right red nucleus
4	Right Substantia nigra
6	Right subthalamic nucleus
8	Right caudate
10	Right putamen
12	Right globus pallidus external
14	Right globus pallidus internal
16	Right thalamus

**Table 6:** Types of Features calculated from the segmented 3D Voxels of Interest.

Feature Type	Number of Features
First Order Statistical Features	17 Features
Shape-Based (2D)	10 Features
Shape-Based (3D)	10 Features
Gray Level Cooccurrence Matrix	24 Features
Gray Level Run Length Matrix	12 Features
Gray Level Size Zone Matrix	12 Features
Neighbouring Gray Tone Difference Matrix	12 Features
Gray Level Dependence Matrix	10 Features
Exhaustive set of Features (Total)	107 Features

**Table 7:** Best set of derived features after applying filter methods and recursive feature elimination.

Feature Name	Description	Equation
Skewness	It measures the asymmetry of the distribution of values about the Mean value.	Skew = $\frac{\mu_{3}}{\sigma_{3}}=\frac{\frac{1}{N_{p}}\sum_{i=1}^{N_{p}}{\left(X\left(i\right)-\bar{X}\right)}^{3}}{{\left(\sqrt{\frac{1}{N_{p}}\sum_{i=1}^{N_{p}}{\left(X\left(i\right)-\bar{X}\right)}^{2}}\right)}^{3}}$
Entropy	It measures the uncertainty or randomness in the image values.	Entropy = $-\sum_{i=1}^{N_{g}}p\left(i\right)log_{2}\left(p\left(i\right)+\;\in \right)$
Total Energy	It is the measure of the magnitude of voxel values in an image.	Total Energy = $V_{voxel}\sum_{i=1}^{N_{p}}{\left(X\left(i\right)+c\right)}^{2}$
Interquartile Range	It measures the percentile of image data present between 25th percent to 75th percent.	IQR = $P_{75}-P_{25}$
Mesh Volume [38]	It measures the Volume of ROI.	$\begin{array}{c} V_{i}=\frac{O_{a_{i}}\cdot \left(O_{b_{i}}\times \;O_{c_{i}}\right)}{6}\\ V=\;\overset{N_{f}}{\underset{i=1}{\sum }}V_{i} \end{array}$
Surface Area	It measures the extrinsic surface are of the ROI	$\begin{array}{c} A_{i}=\;\frac{1}{2}\;\left|a_{i}b_{i}\;\times \;a_{i}c_{i}\right|\\ A=\;\overset{N_{f}}{\underset{i=1}{\sum }}A_{i} \end{array}$
Major Axis Length	It measures the largest axis length of the ROI enclosing ellipsoid using the largest principal component.	Major axis = $4\sqrt{\lambda_{major}}$
Minor Axis Length	It measures the second- largest axis length of the ROI enclosing ellipsoid using the largest principal component.	Minor axis = $4\sqrt{\lambda_{minor}}$
Autocorrelation	It measures the magnitude of the fineness and coarseness of texture.	Auto_corr = $\sum_{i=1}^{N_{g}}\sum_{j=1}^{N_{g}}p\left(i,\;j\right)i$
Cluster Prominence	It measures the skewness and asymmetry of the Gray Level Co-occurrence Matrix.	CP = $\sum_{i=1}^{N_{g}}\sum_{j=1}^{N_{g}}{\left(i+j-\mu_{x}-\mu_{y}\right)}^{4}p\left(i,j\right)$
Difference Entropy	It is a measure of the randomness/variability in neighborhood intensity value differences.	DE = $\sum_{k=0}^{N_{g}-1}p_{x-y}\left(k\right)log_{2}(p_{x-y}\left(k\right)+\in )$
Inverse Difference Moment	IDM measures the local homogeneity of an image.	IDM = $\sum_{k=0}^{N_{g}-1}\frac{p_{x-y}\left(k\right)}{1+k^{2}}$
Small Area High Gray Level Emphasis (SAHGLE)	It measures the proportion in the image of the joint distribution of smaller size zones with higher gray level values	SAHGLE = $\frac{\sum_{i=1}^{N_{g}}\sum_{j=1}^{N_{s}}\frac{P\left(i,\;j\right)i^{2}}{j^{2}}}{N_{z}}$
Gray Level Non-Uniformity [16]	It measures the similarity of gray level intensity values in the images, where a lower GLN value correlates with a greater similarity in intensity values	GLN = $\frac{\sum_{i=1}^{N_{g}}{\left(\sum_{j=1}^{N_{r}}P(i,j|\theta )\right)}^{2}}{N_{r}\left(\theta \right)}$
Gray Level Variance	It measures the variance in gray level intensity for the runs	GLV = $\sum_{i=1}^{N_{g}}\sum_{j=1}^{N_{r}}p\left(i,j|\theta \right){\left(i-\mu \right)}^{2}$
Run Entropy	It measures the randomness in the distribution of run length and gray levels.	RE = $\sum_{i=1}^{N_{g}}\sum_{j=1}^{N_{r}}p\left(i,j|\theta \right)\log_{2}\left(p\left(i,j|\theta \right)+\in \right)$
Long Run High Gray Level Emphasis (LRHGLE)	It measures the joint distribution of long-run lengths with higher gray-level values.	LRHGLRE = $\frac{\sum_{i=1}^{N_{g}}\sum_{j=1}^{N_{r}}P\left(i,j|\theta \right)i^{2}j^{2}}{N_{r}\left(\theta \right)}$
Coarseness	It measures the average difference between the center voxel and its neighborhood and is an indication of the spatial rate of change	Coarseness = $\frac{1}{\sum_{i=1}^{N_{g}}p_{i}s_{i}}$
Small Dependence Emphasis	It measures the small dependencies, with a greater value indicative of smaller dependence and less homogeneous textures.	SDE = $\frac{\sum_{i=1}^{N_{g}}\sum_{j=1}^{N_{d}}\frac{P\left(i,\;j\right)}{i^{2}}}{N_{z}}$
Dependence Non-Uniformity	It measures the similarity of dependence throughout the image, with a lower value indicating more homogeneity among dependencies in the image.	DN = $\frac{\sum_{j=1}^{N_{d}}{\left(\sum_{i=1}^{N_{g}}P\left(i,j\right)\right)}^{2}}{N_{z}}$

**Table 8:** Classifier Hyperparameters.

Classifier	Specification
Artificial Neural Network (MLP)	Layers: 5, Neurons in each Layer: 20, 10, 128, 256, 64, 16, 3, Loss: Categorical Cross-Entropy, Optimizer: AdaDelta, Learning Rate: Start: 1.0—Auto Reduce on Plateau Fraction: 0.8 at 2 simultaneous non decline of validation loss
XgBoost	n_estimators: 600, max_depth = 9, booster: gbtree
Random Forest	n_estimators: 1000, criterion = ‘gini’, max_depth:7, min_samples_split = 20, min_samples_leaf = 10
Support Vector Machine	Kernel = ‘rbf’, degree = 3, gamma = 0.0001 C = 1.0, tol = 0.001, cache_size = 200

**Table 9:** Class Weights.

Classifier	Specification
Control	0.3486
Prodromal	0.8365
Parkinson’s Disease	0.14

**Table 10:** Comparative analysis of the performance of machine learning classifiers and cross-validation results of the classifiers.

Metrics	Test Set	Artificial Neural Network	XgBoost	Random Forest Classifier	Support Vector Machine
Accuracy	Split 1	94.74%	88.23%	85.26%	79.15%
Precision		0.952	0.9025	0.8715	0.8025
Recall		0.9125	0.8947	0.8352	0.7744
F1-Score		0.9655	0.9133	0.8549	0.80
Accuracy	Split 2	95.3%	90.52%	87.32%	81.74%
Precision		0.9728	0.9154	0.9047	0.8241
Recall		0.9541	0.8922	0.8678	0.8071
F1-Score		0.94	0.9257	0.88	0.8178
Accuracy	Split 3	92.8%	87.1%	82.13%	78.83%
Precision		0.9133	0.9068	0.8536	0.8057
Recall		0.9052	0.8534	0.7954	0.772
F1-Score		0.9418	0.8724	0.8204	0.8196
Accuracy	Split 4	93.26%	89.43%	83.6%	80.91%
Precision		0.9145	0.9025	0.8572	0.8133
Recall		0.9254	0.8741	0.8246	0.7924
F1-Score		0.9388	0.8835	0.8391	0.8037
Accuracy	Split 5	89.44%	85.36%	80.44%	76.59%
Precision		0.8832	0.8679	0.8278	0.7964
Recall		0.8728	0.8321	0.7945	0.7512
F1-Score		0.890	0.8522	0.8127	0.7739
